# The Role of β-Arrestins in Regulating Stem Cell Phenotypes in Normal and Tumorigenic Cells

**DOI:** 10.3390/ijms21239310

**Published:** 2020-12-07

**Authors:** Georgios Kallifatidis, Kenza Mamouni, Bal L. Lokeshwar

**Affiliations:** 1 Department of Biological Sciences, Augusta University, Augusta, GA 30912, USA; 2 Georgia Cancer Center, Augusta University, Augusta, GA 30912, USA; KMAMOUNI@augusta.edu; 3 Research Service, Charlie Norwood VA Medical Center, Augusta, GA 30904, USA

**Keywords:** β-arrestin1 (ARRB1), β-arrestin2 (ARRB2), stem cell phenotype, self-renewal, cancer stem cells

## Abstract

β-Arrestins (ARRBs) are ubiquitously expressed scaffold proteins that mediate inactivation of G-protein-coupled receptor signaling, and in certain circumstances, G-protein independent pathways. Intriguingly, the two known ARRBs, β-arrestin1 (ARRB1) and β-Arrestin2 (ARRB2), seem to have opposing functions in regulating signaling cascades in several models in health and disease. Recent evidence suggests that ARRBs are implicated in regulating stem cell maintenance; however, their role, although crucial, is complex, and there is no universal model for ARRB-mediated regulation of stem cell characteristics. For the first time, this review compiles information on the function of ARRBs in stem cell biology and will discuss the role of ARRBs in regulating cell signaling pathways implicated in stem cell maintenance in normal and malignant stem cell populations. Although promising targets for cancer therapy, the ubiquitous nature of ARRBs and the plethora of functions in normal cell biology brings challenges for treatment selectivity. However, recent studies show promising evidence for specifically targeting ARRBs in myeloproliferative neoplasms.

## 1. Introduction

**Figure 1:**
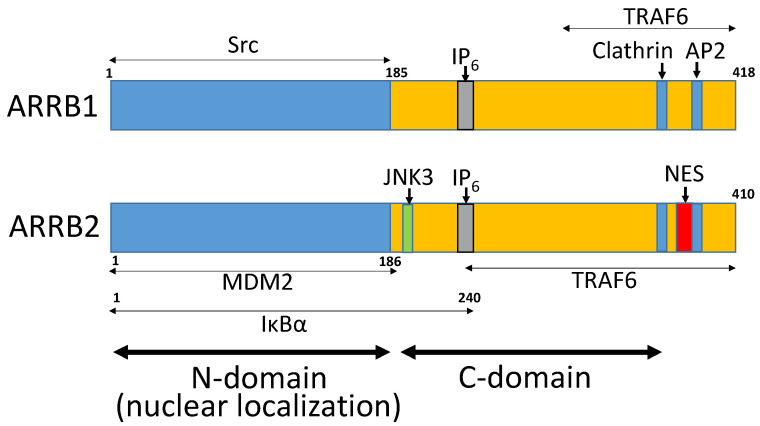
The structure of β-arrestins (ARRBs). Nuclear localization is mediated by the N domain of ARRB1 and ARRB2. A nuclear export signal is located in the C-terminus of ARRB2. The binding sites of various signaling molecules are located in both N and C domains (Figure adapted from Ma and Pei [1]).

Arrestins belong to a family of intracellular proteins that consists of four members. Visual (arrestin 1) and cone arrestins (arrestin 4) are exclusively expressed in the retina, while β-arrestin1/ARRB1 (arrestin 2) and β-arrestin2/ARRB2 (arrestin 3) are ubiquitously expressed in most other tissues [1,2]. β-Arrestins (ARRBs) are cytosolic proteins implicated in the inactivation of activated G-protein-coupled receptor (GPCR) signaling. They translocate to the plasma membrane in the event of ligand-bound activation of GPCRs. ARRB binding to GPCRs uncouples receptors from heterotrimeric G proteins and targets them to clathrin-coated pits for endocytosis. However, in certain circumstances, ARRBs can function as adaptor molecules that mediate G-protein independent signaling by serving as scaffolds that link signaling networks [3]. ARRBs interact with a plethora of cytoplasmic signaling molecules (Figure 1). Importantly, ARRBs regulate mitogenic and anti-apoptotic signaling and play an essential role in tumor vascularization and metastasis [4]. Recent evidence suggests that ARRB1 and ARRB2 regulate stem cell phenotypes in health and disease. The current review focuses on the role of ARRBs in regulating stem cell properties such as self-renewal in normal and cancer stem cells.

## 2. The Role of β-Arrestins (ARRBs) in Normal Stem Cell Maintenance

Dissecting the signaling pathways in adult stem cells will undoubtedly facilitate the development of more effective therapeutic strategies for targeting cancer stem cells (CSCs). In this section, we will review the studies that have confirmed the role of ARRBs in normal stem cell regulation.

### 2.1. Embryonic Stem Cells

Embryonic stem cells (ESCs) are pluripotent cells that give rise to all somatic cell types in the embryo as it develops [5]. Due to their plasticity and potentially unlimited self-renewal capacity, ESCs are proposed for regenerative medicine and tissue replacement after injury or diseases [6]. Lysophospholipid signaling mediators are critical regulators of the development, differentiation, migration, and proliferation of ESCs [7,8]. Among the lysophospholipids, sphingosine-1 phosphate (S1P) exerts various beneficial effects on ESCs, such as promoting ESC migration and proliferation [9]. Although the authors did not specify which ARRB family member was involved, Ryu et al. [10] demonstrated that S1P binding to its receptor (S1P receptor 1 or S1P receptor 3), stimulates ARRB translocation from the cytosol to the plasma membrane and sequentially activates c-Src. It is not clear from the published work whether SIP1-3 activates 7-TM receptors, which signal through ARRB2. In some cases, signal attenuation is achieved through ARRB1/2 [11]. This binding of c-Src and ARRB is mediated in part by an interaction between the c-Src homology (SH) 3 domain of the kinase and proline-rich PXXP motifs in the ARRB [12]. Subsequently, activated ARRBs and c-Src stimulate signaling complex formation between S1PR1/S1PR3and Flk-1 and elicit phosphorylation of Flk-1. Therefore, Flk-1 activation leads to the activation of the downstream targets, including ERK and JNK, which stimulate ECSs proliferation.

### 2.2. Hematopoietic Stem/Progenitor Cells

Hematopoiesis is a dynamic process that involves the interplay between lineage-specific transcription factors and epigenetic regulators. Yue et al. [13] identified ARRB1 as a regulator of hematopoietic lineage specification. Zebrafish embryos depleted of ARRB1 displayed severe posterior defects and notably failed to undergo hematopoiesis. Besides, the expression of cdx4, a critical regulator of embryonic blood formation, and its downstream hox genes were downregulated by depletion of ARRB1, while injection of cdx4, hoxa9a or hoxb4a mRNA rescued the hematopoietic defects. Further mechanistic studies revealed that ARRB1 bound to and sequestered the polycomb group (PcG) recruiter YY1, and relieved PcG-mediated repression of the cdx4-hox pathway, thus regulating hematopoietic lineage specification. Another study revealed the involvement of ARRB2 in the cross-talk between CXCR7 and CXCR4 in response to CXCL12 on primary human CD34^+^ hematopoietic stem/progenitor cells (HSPCs) [14]. The authors found that CXCR7 co-localized with CXCR4 in CD34^+^ cells and that both CXCR7 and CXCR4 mediated the cell cycling promoting effect of CXCL12 on CD34^+^ cells. Interestingly, the incubation with CXCL12 induced the translocation of ARRB2 to the nucleus. This nuclear translocation was inhibited after the neutralization of either CXCR4 or CXCR7 with a specific antagonist. As CXCL12-promoted signaling pathways include the PI3K/Akt pathway [15], the authors investigated whether CXCR7 or CXCR4 could affect Akt phosphorylation in response to CXCL12. They found that CXCL12 increased the expression of phospho-Akt-Ser473 and that blocking either CXCR7 or CXCR4 prevented the CXCL12-induced Akt phosphorylation. Also, a specific siRNA against ARRB2 markedly reduced CXCL12-induced Akt phosphorylation. In conclusion, their results indicated that CXCR7, in cooperation with CXCR4, promotes the survival of HSPCs via the ARRB2-dependent activation of Akt.

Furthermore, studies in ARRB1 and ARRB2 knockout mice have shown that depletion of ARRBs does not affect the relative frequency of hematopoietic stem cell (HSC)-enriched cell populations based on the c-Kit^+^ Lin^−^ Sca-1^+^ (KLS) expression profile [16]. However, depletion of ARRB2 severely impacted HSC functions such as self-renewal and the potential to repopulate mice that were lethally irradiated [16], demonstrating the importance of ARRB2 in regulating pathways implicated in self-renewal.

### 2.3. Neural Precursors

Unlike other members of the arrestin family that appear to be mainly cytoplasmic, ARRB1 is found in both the cytoplasm and the nucleus [17]. ARRB1 can form a nuclear complex with CREB and P300, and this interaction increases specific gene transcription [13]. One of the genes whose transcription was increased by this mechanism was p27^Kip1^, a cyclin-dependent kinase (CDK) inhibitor and differentiation marker for granule neuron precursors (CGNPs) [17,18]. Parathath et al. [19] reported that Sonic hedgehog (Shh) signaling induces ARRB1 accumulation and localization to the nucleus to increase p27 expression and ultimately drive the CGNPs cell cycle exit. In the dentate gyrus (DG) of the adult hippocampus, ARRB1 modulates the proliferation of neural precursors [20]. Indeed, ARRB1 knockout (KO) mice displayed a decrease in neural precursor proliferation. The overexpression of wild type ARRB1 could enhance the cell proliferation in the subgranular zone of ARRB1 KO mice, while nuclear-function-loss Q394L and K157A mutants of ARRB1 could not. RNA-sequencing transcriptome analysis performed in ARRB1 KO astrocytes revealed an elevated transcription of bone morphogenetic protein 2 (BMP2), a well-known anti-mitotic morphogen previously reported to regulate neural precursor proliferation in hippocampi [21]. At both transcription and translation levels, BMP2 was increased in ARRB1 KO astrocytes and the conditioned-media derived from ARRB1 KO astrocytes [20]. Therefore, although the detailed underlying mechanisms need further elucidation, the authors hypothesized that ARRB1 could regulate the transcription of BMP2 in astrocyte-neural precursor interaction to enhance the mitotic expansion of neural precursors in the adult hippocampus.

### 2.4. Mesenchymal Stem Cells

Mesenchymal stem cells (MSCs) are multipotent cells with self-renewal capacity to differentiate into osteogenic, chondrogenic, and adipogenic lineages [22,23]. Human MSCs constitutively produce prostaglandin E_2_ (PGE_2_) that plays a crucial role in hematopoietic stem cell growth and development in embryonic and adult stem cell homeostasis [24]. PGE_2_ exerts its biological action by binding to specific receptors (EP1, EP2, EP3 and EP4) that lead to a variety of downstream events depending on cell type-specific expression patterns [25,26]. Yun et al. [27] reported for the first time that ARRB1 is involved in PGE_2_—induced proliferation and migration in human hMSCs. The authors demonstrated that PGE_2_ leads to phosphorylation of JNK and that a specific siRNA against ARRB1 decreases JNK phosphorylation. Also, a specific siRNA against JNK reduced PGE_2_—induced cell migration and proliferation of human MSCs. To summarize their study, Yun et al. [27] reported that PGE_2_ stimulates migration and proliferation of human MSCs via interaction of Pfn-1 and F-actin through EP2 receptor-dependent ARRB1/JNK pathways.

### 2.5. Cardiac Progenitors

Stem cell-based therapy is investigated as an innovative and promising strategy to restore cardiac structure and function by regenerating functional myocardium [28,29]. Recent evidence indicates that the adult heart is capable of cardiomyocyte turn-over, possibly from endogenous cardiac stem cells/cardiac progenitor cells (CPCs). The ability of CPCs to migrate and form tubes can be an important characteristic to rescue ischemic myocardium [30]. Interestingly, simultaneous knockout (KO) of ARRB1 and ARRB2 caused intrauterine fetal death due to vascular and lymphatic system development failure. Still, mice with a single KO of ARRB1 or ARRB2 did not show apparent abnormality of cardiovascular tissue development [31]. Seo et al. [32] investigated the role of ARRB2 on survival, mobility, and tube formation of cardiac progenitor cells (CPCs) obtained from wild-type (WT) mice (CPC-WT), and ARRB2 KO mice (CPC-KO). They report that compared to CPC-WT, CPC-KO presented significantly worse mobility in the wound healing assay and tube formation on Matrigel under normoxic and hypoxic conditions [32]. Under these findings, CPC-KO demonstrated worse expression profiles of proteins related to cell mobility and tube formation, e.g., protein kinase B (Akt), β-catenin, and glycogen synthase kinase-3β (GSK-3β).

Another research group [33] demonstrated that ARRB2 could promote the differentiation of cardiac stem cells (CSCs) into cardiomyocytes through an ARRB2/miR-155/GSK3β pathway.

### 2.6. Myoblast Cells

During myogenesis, myoblasts fuse into multi-nucleated myotubes to form muscle fibers [34]. Obestatin/GPR39 signaling operates as an autocrine function to control the myogenic program by regulating the different stages involved in myogenesis such as proliferation, migration, fusion, and myofiber growth [35,36]. Obestatin/GPR39 signaling leads to an induction of the Akt/mTOR anabolic growth, which exerts a role in protein synthesis and increases muscle size [35,36,37]. Recent studies unveil a molecular mechanism in which ARRB1 and ARRB2 function as a link between GPR39 and the epidermal growth factor receptor (EGFR) to regulate specific steps of the myogenic process. Obestatin-induced mitogenic action is mediated by ERK1/2 and Jun D activity, in a G-dependent mechanism. At a later stage of myogenesis, ARRB1 and ARRB2 are necessary to activate cell cycle exit and differentiation through JNK/c-jun, CAMKII, Akt and p38 pathways [37].

## 3. The Role of β-Arrestins (ARRBs) in the Maintenance of Cancer Stem Cells

### 3.1. Bladder Cancer

**Figure 2:**
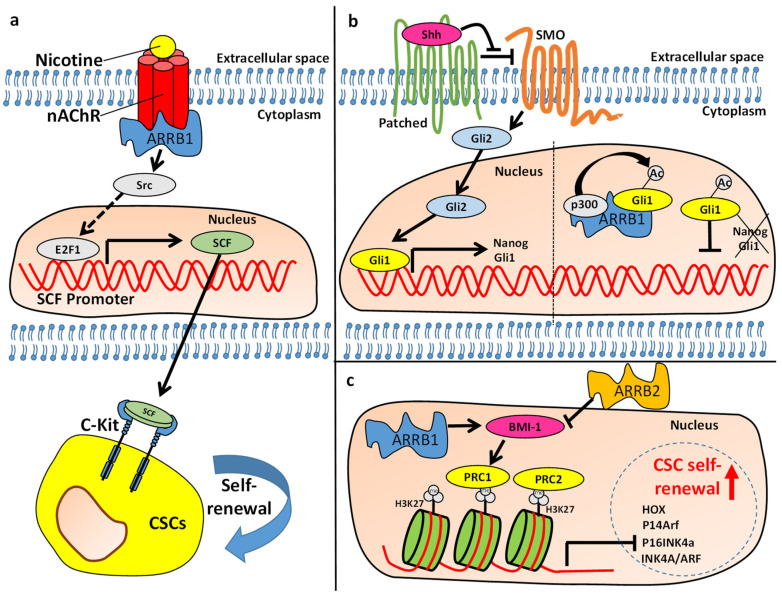
The role of β-arrestins (ARRBs) in regulating self-renewal in solid cancers. (**a**) ARRB1 plays a crucial role in nicotine mediated effects in non-small cell lung cancers (NSCLCs). Binding of nicotine to nicotinic acetylcholine receptors (nAChRs) results in activation of Src in an ARRB1-depended fashion. Src activates the transcription factor E2F1, which drives expression of stem cell factor (SCF) in NSCLC cells. Once SCF is secreted, it can bind c-Kit receptors on cancer stem cells (CSCs) and induce self-renewal. (**b**) When Patched is in a ligand-unbound state, it represses Smoothened (Smo). Binding of Sonic hedgehog (Shh) to Patched results in activation of Smo, which activates transcription factors Gli2/3. The later drive expression of Gli1, which drives expression of genes that regulate self-renewal. ARRB1 promotes the p300-mediated acetylation of Gli1 inhibiting its transcriptional activity and thus expression of genes involved in self-renewal in Shh-medulloblastoma. (**c**) ARRB1 and ARRB2 have opposing effects on Bmi-1 in bladder cancer. ARRB1 positively regulates Bmi-1, a component of the polycomb regulatory complex 1 (PRC1). PRC1 and PRC2 cooperate by binding to trimethylated lysine residues of H3 (H3K27), causing changes in chromatin structure and transcriptional silencing of genes regulating CSC differentiation (e.g., Hox) and self-renewal (Ink4a/Arf) [50,51]. ARRB2 inhibits Bmi-1 mediated self-renewal.

Bladder cancer is a highly heterogeneous disease with a high rate of recurrence. The high recurrence rate is related to both multifocal origin and presence of a heterogeneous mixture of cancer stem cells (CSCs) [38,39]. Identification of bladder CSCs is challenging because of the lack of known, specific markers [40]. However, recent evidence suggests that ARRBs may regulate known stem cell markers and properties in bladder cancers. ARRB1 and ARRB2 appear to have opposing functions in regulating stem cell properties in bladder cancer. While ARRB1 functions as an oncogene and positively regulates expression of stem cell markers, ARRB2 functions as a tumor suppressor and negatively regulates stem cell properties [41]. Studies on bladder cancer cell lines suggest that ARRB2 negatively regulates the cancer stem cell (CSC) marker Aldehyde dehydrogenase (ALDH) [41], which is implicated in the maintenance of CSC stemness and is important for the progression of bladder cancer [40,42]. Similarly, ARRB2 negatively regulates CD44 [41], a marker associated with stem cells and resistance to chemotherapy and metastasis [38,43,44,45]. It also regulates the transcription factor and CSC marker, B-cell-specific Moloney murine leukemia virus insertion site 1 (BMI-1) (Figure 2c) [41]. BMI plays a crucial role in the maintenance of self-renewal in embryonic and adult stem cells [44,46].

ARRB2 also seems to regulate the transcription factor STAT3 in bladder cancer [41]. STAT3 is implicated in a plethora of cellular events, and activation of STAT3 also plays a key role in the malignant transformation of urothelial progenitor cells, the formation of carcinoma in situ (CIS) and progression to the muscle-invasive cancer following treatment with the carcinogen *N*-butyl-*N*-(4-Hydroxybutyl) nitrosamine (BBN) in an animal model [47]. Ho et al. showed that STAT3 signaling enriches KRT14^+^ CSC in CIS, which contributes to the BBN induced progression to invasive tumor formation [47]. Furthermore, STAT3 expression correlates with resistance towards chemotherapy and metastasis. Furthermore, increased expression and activation of STAT3 is crucial in maintaining the self-renewal in bladder CSCs [48,49]. ARRB2 depletion in bladder cancer cells was shown to induce phosphorylation/activation of STAT3, whereas overexpression of ARRB2 had the opposite effect [41].

Previous studies have utilized several markers for isolation of bladder CSCs including CD47, CD90, CD44, cytokeratin 5 (KRT5), KRT14, and KRT17. Recent evidence suggests that CSCs within the basal urothelium are the cells of origin of the muscle invasive bladder cancer. This basal phenotype is characterized by the expression of CSC markers KRT14 and KRT17. Strikingly, ARRB2 was found to negatively regulate both KRT14 and KRT17 in bladder cancer cell lines. Consistently with these observations, overexpression of ARRB2 reduced spheroid formation and viability confirming its role in regulating self-renewal in bladder cancer. Moreover, ARRB2 was shown to regulate resistance of bladder cancer cells towards Gemcitabine. Overexpression of ARRB2 induced expression of ENT1, which promotes influx of Gemcitabine into cancer cells, and was also shown to inhibit conversion of gemcitabine into inactive metabolites by inhibiting the enzyme Cytidine Deaminase (CDA). Importantly, expression of ARRB2 in patient tumor specimens inversely correlated with metastasis and gemcitabine + cisplatin treatment failure.

In contrast, depletion of ARRB1 in bladder cancer cells abrogated expression of CD44 and resulted in significantly reduced protein levels of Bmi1. Consistently, depletion of ARRB1 inhibited self-renewal. Moreover, high levels of ARRB1 in tumors were associated with higher risk of metastasis.

In conclusion, the adapter protein ARRB2 was shown to negatively regulate CSC marker expression, metastasis and resistance towards chemotherapy. However, ARRB1 promotes the metastatic phenotype, including expression of CMC markers and self-renewal. The balance between ARRB1 and ARRB2 may be critical for regulating the maintenance of CSCs in bladder tumors (Figure 2c).

### 3.2. Non-Small Cell Lung Cancer

Smoking is the greatest risk factor for lung cancer and is responsible for the majority of non-small cell lung cancers. Nicotine, which is also the addictive component of tobacco smoke, exerts its effects through nicotinic acetylcholine receptors (nAChRs), which are widespread in neuronal tissues as well as several non-neuronal tissues [52,53,54]. It activates multiple signaling pathways implicated in lung cancer progression. In non-small cell lung cancer (NSCLCs), nicotine stimulation induces mitogenic signaling in an ARRB1 depended fashion [53]. Binding of nicotine to nAChRs recruits ARRB1, which forms an oligomeric complex together with nAChR and Src, which drives proliferation of NSCLC cells [53]. A study conducting microarray analysis in ARRB1-depleted lung adenocarcinoma cell line A549 versus parental A549 cells has shown that nicotine stimulation drives expression of Stem Cell Factor (SCF) in an ARRB1-mediated fashion [55]. Depletion of transcription factor E2F1 decreased nicotine mediated expression of SCF, suggesting that the nicotine binding to its receptor and subsequent recruitment of ARRB1 elicit signaling pathways that activate E2F1 mediated expression of SCF (Figure 2a). SCF and its receptor, c-Kit are essential for maintaining self-renewal in several stem cell types. Experiments conducted in the lung adenocarcinoma cell lines A549 and H1650 have shown that the stem cell-like side population (SP) within these cell lines requires SCF for self-renewal maintenance since abrogation of SCF dramatically diminished nicotine mediated spheroid formation potential [55]. Importantly, depletion of ARRB1 inhibited nicotine-mediated SCF expression and SCF-mediated self-renewal in the SP [55]. Consistently, over expression of ARRB1 increased the fraction of the SP in A549 cells, whereas depletion of ARRB1 resulted in an decrease of the SP fraction in several NSCLC cell lines [56]. Moreover, overexpression of ARRB1 resulted in an increase in the protein level of the ATP-binding cassette (ABC) transporter ABCG2, a marker associated with stem cell populations and the ability of the SP to efflux dyes and drugs [56]. In contrast, ARRB2 played only a marginal role in regulating the SP [56].

In conclusion, ARRB1 positively regulates stem cell properties such as self-renewal in the SP of NSCLC cells.

### 3.3. Leukemia

Chronic myelogenous leukemia (CML) is caused by a gene translocation of BCR and ABL genes resulting in the fusion oncogene BCR-ABL. The latter is responsible for the dysregulated activation of the tyrosine kinase ABL [16]. Research studies on HSC-enriched cell populations have shown that HSCs from ARRB2 knockout mice have a significantly reduced self-renewal potential compared to HSCs derived from wild-type control mice [16]. When HSC-enriched populations were isolated from ARRB2 depleted mice and transduced with the p210 form of the BCL-ABL fusion oncogene for recapitulation of CML in mice, they failed to establish CML in 93% of wild-type recipient mice, suggesting that ARRB2 plays a key role in initiation of CML [16]. Furthermore, depletion of ARRB2 in sorted CML stem cells resulted in reduced numbers of colonies when cells were serially passaged, suggesting that ARRB2 regulates self-renewal in CML stem cells.

Mechanistically, ARRBs are implicated in the Wnt/Frizzled pathway and play a role in activation of β-catenin [57]. The WNT/Frizzled pathway is crucial for embryonic development, and deregulation of this pathway is implicated in developmental defects and diseases [58]. For instance, aberrant/consistent activation of the WNT/Frizzled pathway is a crucial step in the genesis of a variety of cancers [57,58]. Experiments on HSC-enriched KLS cells have demonstrated that transformation with BCR-ABL results in activation of β-catenin. Importantly, depletion of ARRB2 significantly reduced the level of activated β-catenin suggesting that ARRB2 also plays a key role in regulating the Wnt/Frizzled pathway in CML [16]. Moreover, ARRB2 is also implicated in the more aggressive blast-crisis phase (bcCML) of CML in which blast cells exhibit severely impaired differentiation and increased proliferation. Depletion of ARRB2 (in contrast to ARRB1) abrogated self-renewal of bcCML cancer stem cells and promoted differentiation as shown in in serial transplantation assays in mice [16]. In conclusion, ARRB2 activates β-catenin in CML and bcCML and plays a crucial role in regulating self-renewal and differentiation in leukemic cancer stem cell populations promoting initiation and progression of the disease.

**Figure 3:**
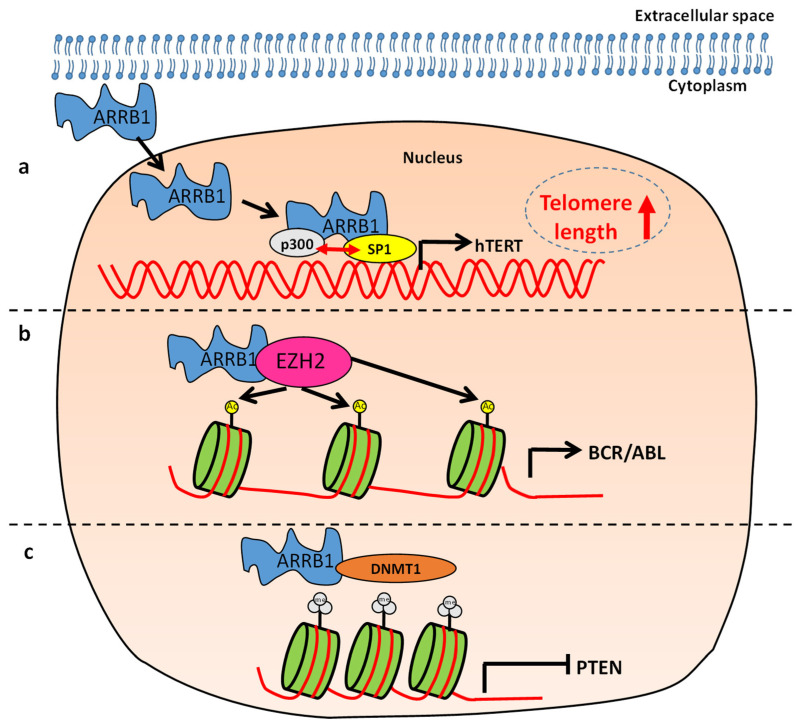
The role of β-arrestin1 (ARRB1) in regulating self-renewal and progression of myeloproliferative neoplasms. (**a**) ARRB1 promotes p300-SP1 interaction and SP1 mediated transcription of human telomerase reverse transcriptase (hTERT). Thus, ARRB1 is important for telomerase activity preventing the onset of cellular senescence in leukemia stem cells (LSCs). (**b**) ARRB1 facilitates enhancer of zeste homologue 2 (EZH2)-mediated acetylation of BCR/ABL histone H4, promoting progression of chronic myeloid leukemia. (**c**) ARRB1 facilitates the DNMT1-mediated methylation of the PTEN promoter region, promoting epigenetic silencing of PTEN and generation of leukemia stem cells in B-lineage acute lymphoblastic leukemia.

The role of ARRB1 in progression of CML is controversial. Fereshteh et al. claim that ARRB1 is not required for propagation of CML in vivo. In contrast, Li et al. show that ARRB1 expression promotes progression of CML and its expression levels increase as CML progresses from an early chronic phase (CP) through an accelerated phase (AP) to a blast crisis phase (BC). Moreover, the authors show that ARRB1 promotes proliferation of CML cell line K652 in vitro and well as primary CML cells. Consistently, depletion of ARRB1 prolonged survival of CML mice [59]. Interestingly, ARRB1 was shown to interact with epigenetic regulators such as the enhancer of zeste homologue 2 (EZH2) [59] which plays a role in epigenetic silencing [60]. The ARRB1-EZH2 scaffold complex is recruited to the BCR/ABL promotor regions and promotes progression of CML by regulating histone H4 acetylation (Figure 3b) [59]. Acetylation of histone H3 and histone H4 results in chromatin remodeling and a more open chromatin structure, enabling recruitment of transcription factors and modulators necessary for the transcription of target genes [61]. Further evidence is needed to clarify whether ARRB1 is essential for the EZH2 mediated chromatin remodeling. In conclusion, there is evidence that nuclear ARRB1 interacts with EZH2 to promote acetylation of histone H4 and expression of BCR/ABL promoting progression of CML.

ARRB1 also regulates self-renewal in the cancer stem cell population in B-lineage acute lymphoblastic leukemia (B-ALL), which predominantly affects B cells and approximately accounts for 70% of childhood acute lymphoblastic leukemia (ALL) cases [62]. Shu et al. demonstrated that the CD34+CD38-CD19+ population derived from B-ALL has increased self-renewal as shown by increased potential to form colonies when serially passaged [62]. Furthermore, the aggressiveness of CD34+CD38-CD19+ was confirmed in in vivo studies where the authors demonstrated that transplantation of NOD/SCID mice with these cell population decreased animal survival significantly compared to transplantation with other cell populations. Importantly, this cell population is also characterized by increased expression of both ARRB1 and ARRB2. Consistently, depletion of ARRB1 in these leukemia stem cell (LSC) populations resulted in reduced numbers of primary and secondary colonies in vitro and most importantly, mice transplanted with ARRB1-depleted LSC showed prolonged survival compared to control animals transplanted LSC expressing ARRB1 [62]. These results underline the importance of ARRB1 in regulating self-renewal of LSCs in B-ALL.

Mechanistically, ARRB1-mediated epigenetic regulation of the tumor suppressor PTEN plays a key role in maintaining the self-renewal potential in the cancer stem cell-enriched CD34+CD38-CD19+ population of B-ALL [62]. Epigenetic silencing of tumor suppressors such as PTEN is a crucial event in initiation of leukemia [63,64] and is often mediated by hypermethylation of CpG rich regions catalyzed by methyltransferases such as DNMT1, DNMT3A and DNMT3B [65,66]. In B-ALL patient-derived LSCs, DNA methylation of PTEN was increased, whereas PTEN expression was decreased compared to other cell populations [62]. Experiments with ARRB1 depleted clones suggested that ARRB1 promotes hypermethylation of PTEN DNA regions by increasing DNMT1 activity. These events resulted in ARRB1-mediated epigenetic silencing of PTEN (Figure 3c) and regulated the self-renewal potential of LSCs [62]. One potential mechanism for ARRB1-mediated activation of DNMT1 could be the interaction of ARRB1 with EZH2, which is known to recruit and activate DNMT1 [67].

Besides its effects on proliferation and self-renewal in LSCs, ARRB1 was also shown to regulate cellular senescence in ALL [68]. Cellular senescence is defined as an irreversible, terminal growth arrest which can be induced by stress-mediated epigenetic changes and/or aging-related telomere shortening. Stem cells exhibit epigenetic changes that prevent the onset of cellular senescence and thus maintain the self-renewal potential. One of the key regulators of self-renewal in several types of cancer including leukemia is Bmi1. Bmi1 has been shown to prevent senescence indirectly, by repressing genes that promote senescence [69]. While the role of ARRB1 in regulating Bmi-1 in leukemia has not been established, there is evidence that ARRB1 is essential for maintaining Bmi1 driven self-renewal in bladder cancer [41]. Nevertheless, ARRB1 plays a role in regulating senescence in ALL by dictating epigenetic changes that promote telomerase activity. Specifically, ARRB1 was shown to mediate interaction of the transcription factor SP1 with the histone acetyltransferase P300 [68]. One of the target genes of SP1, human telomerase reverse transcriptase (hTERT), is crucial for telomerase activity in cancer [68]. ARRB1 mediated recruitment of P300 facilitates chromatin remodeling and SP1 driven expression of hTERT (Figure 3a). These events enhance telomerase activity increasing telomere length and preventing the onset of cellular senescence in ALL [68].

ARRB2 is also critical in regulating signaling in another myeloproliferative disorder, primary myelofibrosis (PMF). PMF is closely related to CML and has a significant mortality rate. About 5–10% of patients with PMF exhibit activating mutations (MPLW515L) of the myeloproliferative leukemia virus (MPL) oncogene which encodes the thrombopoetin receptor [70,71]. Rein et al. demonstrated that ARRB2 plays a crucial role in MPLW515L induced PMF [71]. The authors transduced wildtype and ARRB2-depleted HSCs with MPL-mutant (MPLW515L) retrovirus and investigated the potential of these cells to form PMF in recipient mice. While ARRB2 depleted HSCs exhibited homing to the marrow, they failed to repopulate because of reduced self-renewal potential in contrast to wildtype HSCs [71]. Importantly, ARRB2 depleted HSCs were characterized by increased apoptosis compared to wildtype cells [71]. These data indicate that ARRB2 has anti-apoptotic function in MPLW515L transduced HSC and is also essential self-renewal suggesting a key role of ARRB2 in progression of PMF.

The crucial role of ARRB2 in the onset and maintenance of myeloproliferative neoplasms suggests that it could serve as a promising target for therapy. However, the selective targeting of ARRB2 in cancer cells presents challenges because of its ubiquitous nature. A resent publication utilized aptamer oligonucleotides whose tertiary structure selectively binds and targets ARRB2 in leukemic cells [72]. The authors developed chimeric aptamers by linking ARRB2 specific aptamers to aptamers that selectively bind the cancer cell specific cell membrane antigen nucleolin. They showed that the chimeric aptamers were internalized in cancer cells following binding to nucleolin and interacted with ARRB2 to inhibit ARRB2 mediated scaffolding and activation of ARRB2 mediated Wingless/Frizzled and Hedgehog/Smoothened signaling. Importantly, the chimeric nucleolin/ARRB2 aptamers inhibited clonogenicity in patient-derived leukemic cells [72].

In conclusion, ARRBs play a crucial role in maintaining stem cell properties in leukemia (Figure 3). They regulate proliferation and self-renewal and there is evidence that ARRB1 also prevents the onset of cellular senescence in LSCs. However, the regulation of self-renewal by ARRB1 and ARRB2 differs mechanistically. There is evidence that ARRB2 regulates the Wnt/Frizzled pathway and activates β-catenin signaling. While this pathway may also be activated by ARRB1, the latter also plays a crucial role in regulating epigenetic changes associated with the stem cell phenotype (Figure 3). The lack of epigenetic regulation by ARRB2 could be attributed to its cellular localization. ARRB2 includes a nuclear export signal (NES) and is pronominally located in the cytoplasm in contrast to ARRB1, which lacks an NES and is located in the nucleus in addition to the cytoplasm [73,74]. ARRBs could serve as targets for CSC therapies. ARRB2 targeting aptamers have been utilized to specifically target ARRB2 signaling in leukemic cells.

### 3.4. Medulloblastoma

Medulloblastoma is the most common malignant brain tumor in children, and evidence suggests that it derives from malignant transformation of neural stem cells [75,76]. It is subdivided into five subgroups based on differences at the molecular level. These subgroups include Wingless-type MMTV integration site family (WNT) activated, Sonic Hedgehog (SHH) activated/TP53 mutant, SHH activated/TP53 wildtype, non-WNT/non-SHH/Group3 and non-WNT/non-SHH/Group4 [77]. SHH-driven medulloblastoma is characterized by altered SHH/Gli signaling which regulates stem cell properties in CSCs [78]. Interestingly, ARRB1 and its intragenic miR-326 (located within ARRB1 gene) are down-regulated in SHH medulloblastoma derived CSCs [78]. Overexpression of miR-326 in CSCs inhibited SHH/Gli signaling and expression of target genes as well as spheroid formation/self-renewal. Specifically, miR-326 repressed mRNAs of Smo and Gli2 thus targeting SHH/Gli signaling at the receptor and transcription factor level. Similarly, ARRB1 overexpression in SHH-medulloblastoma CSCs resulted in decreased Gli1 protein levels [78]. Moreover, when CSCs were cultured in media that induced differentiation, the ARRB1 levels were increased, whereas Gli1 was decreased. ARRB1 was shown to form a complex with p300 and Gli1 and to promote the p300-mediated actetylation of Gli1, inhibiting its transcriptional activity and thus expression of stem cell-related target genes (Figure 2b) [78].

Taken together, miR-326 and its host protein, ARRB1, function together to negatively regulate SHH/Gli signaling in medulloblastoma CSCs in a complementary manner. It is crucial for CSC to maintain low levels of ARRB1/miR-326 for maintenance of self-renewal. It is noteworthy that ARRB1 functions as a tumor suppressor in medulloblastoma which is in contrast with its role in other cancers such as bladder cancer and leukemia.

## 4. Targeting β-Arrestins (ARRBs) as a Therapeutic Strategy

Given that ARRBs can activate multiple signaling pathways with distinct or even opposite effects on cell function, strategies to target them directly pose significant challenges. One of the unique features of the ARRBs that makes it difficult to selectively target one isoform is their high level of homology. Despite these difficulties, targeting ARRBs is beginning to emerge as a niche field from a therapeutic standpoint. Several proof-of-principle studies have described strategies to target ARRB functions in a cellular context [79].

A combination of a screening approach and cell-based assays identified a small molecule compound named Barbadin capable of inhibiting ARRB2-β2-adaptin interaction in the cellular context [80]. Barbadin selectively inhibits the interaction between ARRB and the β2-adaptin subunit of the clathrin adaptor protein AP2 without interfering with the formation of receptor/ARRB complexes. The selective ARRB/β2-adaptin inhibitor barbadin blocks agonist-promoted endocytosis of the prototypical β2-adrenergic (β2AR), V2-vasopressin (V2R) and angiotensin-II type-1 (AT1R) receptors, but does not affect ARRB-independent (transferrin) or AP2-independent (endothelin-A) receptor internalization [80].

Another study used an in vitro platform referred to as “Systematic Evolution of Ligands by Exponential enrichment (SELEX)” for selecting RNA aptamers against purified ARRB2 in the context of chronic myelogenous leukemia (CML) [72]. The delivery of the ARRB2-targeting aptamer inhibits multiple ARRB-mediated signaling pathways known to be required for chronic myelogenous leukemia (CML) disease progression, and impairs tumorigenic growth in CML patient samples [72].

From a phage display library, Ghosh et al. isolated and characterized a set of synthetic antibody fragments (FABs) against ARRB isoforms in terms of their selectivity against ARRBs isoforms and their ability to modulate different interactions of ARRBs [81]. One of these FABs, selectively inhibited the interaction of ARRB2 with the clathrin terminal domain in vitro [81]. Upon expression as an intrabody, this particular FAB, now referred to as intrabody5, significantly inhibited agonist-induced endocytosis of a diverse set of GPCRs [81]. Other approaches to modulate ARRB interaction and its ensuing functional outcome include the generation of nanobodies and monobodies [82].

Finally, another avenue to consider is to modulate ARRBs functions through biased agonism at GPCRs. Biased agonists stabilize receptor conformations preferentially stimulating one pathway over others, allowing a more targeted modulation of cell function and treatment of disease [83].

## 5. Conclusions

ARRBs are negative regulators of G-protein-coupled receptor (GPCR) signaling and are also known to function as scaffolds that link signaling networks, thus mediating G-protein independent signaling in health and disease. Increasing evidence suggests that ARRBs regulate stem cell properties such as self-renewal in non-malignant stem cells as well as CSCs. There is no universal model for ARRB-mediated regulation of stem cell characteristics. ARRB1 predominantly functions as an oncogene that positively regulates self-renewal in CSCs with the exception of medulloblastoma CSCs where ARRB1 inhibits SHH/Gli signaling and expression of target genes associated with stemness. ARRB2 functions as a negative regulator of stem cell properties in bladder cancer, however, it induces self-renewal in leukemia where it plays a crucial role in stem cell maintenance. ARRBs are potential targets for cancer therapy. Many cancers are driven from CSCs and both ARRB1 and ARRB2 were shown to be crucial in regulating CSC self-renewal (Table 1). Many tumors are highly heterogeneous and there is no universal marker to identify CSCs. However, CSCs are universally characterized by the ability to self-renew, thus targeting ARRBs is a promising therapeutic strategy. The ubiquitous nature of ARRBs and plethora of functions in normal cell biology brings challenges with treatment selectivity. However, recent attempts with ARRB2 specific aptamers that also specifically bind to cancer cells show promising results for specifically targeting ARRBs in cancer cells and or CSCs.

## Figures and Tables

**Table 1:** The role of β-arrestins (ARRBs) in regulating cancer stem cells (CSCs).

Cancer	Role of ARRB1	Role of ARRB2	Reference No.
Bladder	Stimulates growth and stem-cell phenotype/CSC-self-renewal	Inhibits growth and CSC phenotype, inhibits motility and invasion	[41]
Non-small cell lung cancer	Promotes nicotine-mediated self-renewal of CSCs	-	[55]
Myeloproliferative neoplasms (e.g., leukemia)	Inhibits senescence, promotes self-renewal of CSCs, promotes progression	Anti-apoptotic effects, promotes CSC maintenance, mediates initiation and progression of disease	[16,59,62,68,71]
Medulloblastoma	Inhibits self-renewal of CSCs	-	[78]

## Abbreviations

ARRB1	β-Arrestin1
ARRB2	β-Arrestin2
CSC	Cancer stem cells
ESC	Embryonic stem cells
GPCR	G-protein-coupled receptor
